# Deciphering the mechanisms of phonological therapy in jargon aphasia

**DOI:** 10.1111/1460-6984.12437

**Published:** 2018-11-26

**Authors:** Arpita Bose, Fiona Höbler, Douglas Saddy

**Affiliations:** ^1^ School of Psychology and Clinical Language Sciences University of Reading Reading UK; ^2^ Department of Speech–Language Pathology University of Toronto Toronto ON Canada; ^3^ Department of Research Toronto Rehabilitation Institute Toronto ON Canada

**Keywords:** therapy, cueing, naming, phonology, jargon aphasia, nonword

## Abstract

**Background:**

Severe word production difficulties remain one of the most challenging clinical symptoms to treat in individuals with jargon aphasia. Clinically, it is important to determine why some individuals with jargon aphasia improve following therapy when others do not. We report a therapy study with AM, an individual with severe neologistic jargon aphasia, and provide a subsequent comparison with previous cases, with the purpose of informing both our theoretical and clinical understanding of jargon aphasia.

**Aims:**

To investigate AM's locus of word production deficit and determine the effectiveness of phonological component analysis (PCA) therapy, a phonological cueing therapy, in the re‐learning and generalization of naming responses for words. In addition, AM's performance in therapy, linguistic profile and ability to engage with therapy/cues were compared in a retrospective analysis with the background linguistic and therapy data of two other individuals with jargon aphasia (P9 and FF), who responded differentially to PCA. This was undertake to explore possible prognostic indicators of phonological therapy for jargon aphasia.

**Methods & Procedures:**

A battery of linguistic and neuropsychological tests was used to identify AM's word production deficit. A single‐subject multiple probe design across behaviours was employed to evaluate the effects of PCA therapy on the re‐learning and generalization of naming responses. In the retrospective analysis of AM, P9 and FF, we compared differences and similarities in performance on various linguistic tasks, the ability to engage in therapy (i.e., ability to generate and use the cues), as well as to retain and maintain cues.

**Outcomes & Results:**

AM's locus of deficit was identified in the mapping between semantics and phonology. PCA was found to be effective in improving naming in two of the three treated word lists during the treatment phase; however, these gains were not maintained. Generalization to untreated picture names was not observed. Findings from the retrospective analysis illustrated that oral reading skills, the ability to segment phonological information from words and active engagement with provided cues are likely prerequisites for obtaining robust and long‐term gains.

**Conclusions & Implications:**

We demonstrated that phonological therapy could be beneficial for the remediation of naming abilities at least in the re‐learning phase; however, maintenance and generalization of these gains were limited. This research helps to elucidate the considerations and evaluations necessary for the appropriateness of phonological therapy and candidacy of individuals with jargon aphasia for this treatment approach.

**What this paper adds**

**What is already known on the subject**

Severe word production difficulties remain one of the most challenging clinical symptoms to treat in individuals with jargon aphasia, with some individuals improving after therapy, whilst others do not.

**What this paper adds to existing knowledge**

An important contribution of this research is that it adds to the clinical literature of an under‐researched condition. This study identified the locus of deficit in an individual with jargon aphasia, AM, in the connection from semantics to phonology, and contributed to the evidence of usefulness of phonological therapy in improving naming responses during treatment. However, AM's therapy gains did not generalize to untreated items nor were they maintained in the long term.

**What are the potential or actual clinical implications of this work?**

By providing a comparison with previously reported cases, we offer insight into the possible performance prerequisites required for the successful treatment outcomes using this approach. We provide recommendations for addressing many of the outstanding issues in the therapy literature in jargon aphasia, including: the systematic investigation of the underlying word production deficits in jargon aphasia in relation to therapy efficacy; a detailed understanding of the manner in which different types of cues are processed by individuals that can influence therapy outcomes; the influence of cognitive and learning abilities on therapy gains; as well as developing better means of documenting change to potentially quantify therapy outcomes more clearly.

## Introduction

Jargon aphasia describes the profile of individuals who produce fluent, but largely unintelligible, output often comprised of a high proportion of nonword errors, either interspersed with real words or as a predominant form of output (see Marshall 2006, 2018 for reviews on jargon aphasia). Jargon aphasia is often associated with Wernicke's, transcortical sensory or conduction aphasia (e.g., Marshall 2006, 2018, Moses *et al*. 2004). While substantial research has been devoted to understanding the basis of the output profile in jargon aphasia, these individuals still present both theoretical and clinical challenges. Theoretically, we need to identify the level of deficit within the word production system that explains the impairment. Clinically, we need to develop appropriate interventions to treat the deficit. Marshall (2006) pointed out that jargon aphasia remains ‘one of the most puzzling and clinically intractable forms of aphasia’ (387). Indeed, successful therapies for this population have not been swiftly forthcoming (for a discussion, see Marshall 2018 and Marshall *et al*. 2001).

This research presents two investigations. First, we conducted a detailed investigation to determine the underlying word production deficits in AM, an individual with severe neologistic jargon aphasia, and present the findings from therapy targeted at improving his naming abilities and generalization of naming responses. Second, AM's performance in therapy, linguistic profile and the ability to engage with therapy/cues (i.e., ability to generate and use the cues) were compared in a retrospective analysis with background linguistic and therapy data of two other individuals with jargon aphasia (P9, Leonard *et al*. 2008; FF, Bose 2013). As previously reported in two standalone investigations, these two individuals (P9 and FF) had undergone the same phonological therapy as AM with similar frequency and dosage, but had responded differently. Specifically, P9 did not show any positive gains, whilst FF showed improved naming and maintained some of their gains. The comparison of these three individuals with jargon aphasia on how they engaged with the therapy (i.e., ability to generate and use the cues) has not been reported in the literature to date. This has provided a unique opportunity for the exploration of the possible determiners of success in phonological therapy for jargon aphasia.

Individuals with jargon aphasia show a number of common characteristics, along with considerable heterogeneity (Marshall 2006, 2018). The most salient feature of productive language is fluent output with a high proportion of nonwords (Bose 2013, Olson *et al*. 2007, Robson *et al*. 2003). The nonwords are usually phonologically related to the target; however, nonwords with no clear phonological overlap with the target have also been reported (Eaton *et al*. 2011). Research investigating naming impairments in jargon aphasia has honed in on a deficit either at the phonological level and/or in accessing the phonological level (e.g., Bose and Buchanan 2007, Hillis *et al*. 1999, Kohn *et al*. 1996, Olson *et al*. 2007, Schwartz *et al*. 2004). Robson *et al*. (2003) predict that for jargon aphasia, ‘strengthening the signal to the phonological level will improve output in predictable ways’ (122). Not surprisingly, in some cases of jargon aphasia, therapy and cueing studies using phonological cueing methods have shown to be effective in improving naming and decreasing nonword production (Bose 2013, Bose and Buchanan 2007, Robson *et al*. 1998a).

Only three published research articles address the effectiveness of the techniques aimed at improving spoken naming abilities in jargon aphasia, with equivocal results (Bose 2013, Marshall *et al*. 1998, Robson *et al*. 1998a; see Marshall 2018 for a review of therapy studies). In general, it appears that for jargon aphasia phonological approaches are more beneficial than semantic approaches (e.g., Bose 2013, Marshall *et al*. 1998, Robson *et al*. 1998). Previous research that has used phonological approaches for improving naming abilities in jargon aphasia has included syllable length and initial phoneme judgment tasks (Robson *et al*. 1998), as well as phonological component analysis (PCA) (Bose 2013). The effectiveness of PCA to improve naming in jargon aphasia remains inconclusive; it has previously been shown to be beneficial in improving naming for FF (Bose 2013), but not for P9 (Leonard *et al*. 2008).^1^ This research provides an opportunity to investigate whether the effects of PCA for improving naming in jargon aphasia can be replicated, and thereby build on the evidence of the effectiveness of PCA (or lack thereof). In the existing naming therapy literature for jargon aphasia, gains on naming abilities have been moderate and mostly limited to the trained items, with minimal to no generalization to untreated items.

Marshall (2018) recommended that evaluation of anomia therapy in jargon aphasia is needed to explore the factors that make individuals suitable candidates for therapy or less so. Considering the heterogeneity of participant profiles in jargon aphasia, large‐scale therapy studies with several individuals matched for underlying profiles, therapy approaches, tasks and dosage would ideally be the most informative approach; however, such undertakings are resource intensive. Keeping AM's therapy identical to FF and P9's therapy allowed us to undertake an additional retrospective analysis in which we compared these three participants’ performances in generation and use of phonological cues. This provided an indication of how individuals responded to and generated therapy cues, and if this influences treatment gains and maintenance thereof. This fulfils an important clinical goal of finding possible determiners of success for therapy.

We implemented PCA to ameliorate AM's naming difficulties, and to measure generalization of naming responses to untrained items. In addition to our goal to replicate the effect of PCA on naming in jargon aphasia, we choose PCA for AM because it focuses on the active participation of the client in the generation of target phonological properties or making active choices for phonological components of the target words. This active choice along with cued responses is argued to contribute to deeper processing of the task at hand and to longer‐lasting effects (Hickin *et al*. 2002).

Before implementing therapy with AM, we characterized his word production impairments and compared his naming performance on semantic and phonological cueing tasks. Improved accuracy in the phonological cueing condition would provide evidence that increasing phonological activation is a potential therapeutic avenue to improve naming for AM (Robson *et al*. 2003). As it is not straightforward to localize the point of breakdown in an individual's word production system, we sought information from different sources (see Howard and Gatehouse 2006 for a discussion). We undertook a detailed linguistic investigation of various levels of word processing and production, along with analysis of error profiles and phonological relatedness between target and nonword responses (e.g., Howard and Gatehouse 2006, Robson *et al*. 2003). In the first section of this paper, we investigated the level of word production deficit in AM, implemented PCA, a phonological therapy targeted to improve his naming abilities, and measured generalization of his naming responses to untrained items. In the second section, we performed a retrospective analysis of AM, FF and P9, who responded differentially to PCA, in an attempt to determine the prognostic indicators for successful candidacy in using phonological therapies in jargon aphasia.

The specific research questions were:

- To investigate the locus of deficit for AM within the word production system.
- To assess whether PCA results in re‐learning (trained items) and response generalization (untrained items) of correct naming responses for a set of nouns for AM.

## Methods 1

### Participant and background testing

AM was an 85‐year‐old right‐handed, monolingual speaker of English with 12 years of formal education. He was a retired sales manager. At the time of this research, AM was 15 years post‐onset to two left cerebrovascular attacks, which occurred within a month of each other. He had corrected vision, and reported no previous history of neurological and/or psychiatric disorders. He was not receiving any speech–language therapy at the time of the study. Based on the Boston Diagnostic Aphasia Examination (BDAE; Goodglass *et al*. 2001), AM exhibited a profile of fluent conduction aphasia with level 2 severity. Table 1 presents the BDAE raw scores with percentile ranking across each domain of testing along with connected speech samples. Based on Marshall (2006, 2018), AM showed the characteristics of an individual with neologistic jargon aphasia. He had fluent output with long and relatively grammatical sentences, which were comprised of high proportions of nonwords, and had difficulties in both spontaneous speech and structured speech production tasks. He presented with auditory comprehension difficulties. In word‐production tasks (e.g., picture naming, repetition), he showed severe impairments and produced a high proportion of nonword errors with little phonological overlap to their targets, particularly on the picture‐naming task (see the results from the word‐production tasks below).

**Table 1:** Result from AM's performance on the Boston Diagnostic Aphasia Examination (BDAE, Goodglass *et al*. 2001) and samples of connected speech

		AM's performance	
BDAE (short form)	Total number of items	Raw score	Percentile
Aphasia type		Conduction	
Severity		2	50th
*Speech fluency (seven‐point rating scale)*			
Phrase length		4.5	30th
Melodic line		2	10th
Grammatical form		3.5	30th
Articulatory agility		5	50th
Recitation‐automatized sequence	4	2	20th
*Auditory comprehension*			
Word comprehension	16	15	60th
Commands	10	9	70th
Complex ideational material	6	1	10th
*Repetition*			
Words	5	1	10th
Sentences	2	1	60th
*Naming*			
Responsive naming	10	10	100th
Boston naming test (short form)	15	2	30th
Special categories	12	11	50th

#### Cookie theft picture description from the BDAE

This…children are creeped up to take to take a /kuku/ /khæn/ /tʃænhiː/…a callinɡ, passing it down to the sister and her mother is doing the waiting, and is /brɪwɪŋ/ all the water…./fɪmɪŋ/ her shoes up…plating /brɛdsɪn/…and the window is open for some sun….and curtains here that have been announced…men hose /kəliːlɪŋ/…and he's a fall on the floor…seems that him I think….water's gone…and I think we've said it more or less.

#### Conversational sample with the second author, FH, where AM was asked about his weekend, during which he watched the Queen of England's Jubilee celebrations

FH: So, could you tell me a little bit about what you did this weekend?
AM: This weekend, hmm…I watched the /mægæziːn/ on the /hɛmp/…and I watched it all the time. It's lovely.
FH: Was that the television that you…
AM: Yes.
FH: And, what were they showing?
AM: /twiːn/, /twiːm/, /twiːn/ is coming…and she was tooting herself…a /tɛləm/…made her /swiː/, /tɪdəhʌl/.
FH: And what else did she do?
AM: She /kɛmiə/ the church…and spoke to her…giving presents for us…
FH: And did she give a speech as well?
AM: Yes, she did.
FH: And what did you like about the Jubilee?
AM: It's such a lovely time…it's /fwɪn/ praying…and everyone was so proud of her…preach of her…she was so good.

### Tasks to identify impaired and preserved processes to localize AM's deficit

A comprehensive evaluation of AM's receptive and production processes underlying single word production was performed using various subtests from the Psycholinguistic Assessments of Language Processing in Aphasia (PALPA; Kay *et al*. 1992), the three‐picture version of the Pyramids and Palm Trees Test (PPT; Howard and Patterson 1992) and the Philadelphia Naming Test (PNT; Roach *et al*. 1996). Input and output phonology was characterized by the PALPA subtests: 2 (Real‐word minimal pair discrimination), 4 (Minimal pair requiring picture selection), 5 (Auditory lexical decision), 9 (Word repetition: imageability and frequency), 7 (Word repetition: syllable length), and 8 (Nonword repetition: syllable length). Conceptual and lexico‐semantic processing was characterized using: the three‐picture version of the PPT test, PALPA subtests 47 (Spoken word‐to‐picture matching), 48 (Written word‐to‐picture matching) and 49 (Auditory synonym judgment). Following the suggestion of Marshall *et al*. 1998), we had tested AM's delayed monitoring ability. AM was verbally presented with his own responses to the PALPA 53 naming subtest on a subsequent day to testing, and was asked to judge if those responses were the correct names for the images. With the exception of the delayed monitoring task (performed 1 year prior), all background testing was completed within 2 months before the start of the therapy.

AM's naming responses from the PNT were used to investigate his error profile and the quality of his nonword responses. We followed the standard procedure for PNT administration (Roach *et al*. 1996). The first complete response was identified. Although individuals with jargon aphasia often produce extensive output, studies rarely specify how completed attempts are identified. Roach *et al*. (1996) provide some criteria for identifying the first complete response; ignoring incomplete items such as single phonemes or consonant + schwa and filler items (e.g., um, uh) altogether. Incomplete items were judged on the basis of auditory cues (e.g., segment duration, a lack of downward or questioning intonation, no pause separating an item from the following attempt) which indicated self‐interruption. We provide the following examples to illustrate how the first complete response could be identified, if responses included incomplete items.

Target: vase

Response with single phonemes before first complete: /v/ /vez/

Response with consonant + schwa before first complete: /fə/ /vez/

Response with filler items before first complete: *uh* /vez/

Self‐corrections were not allowed if they followed a complete response. All incorrect responses were scored as errors and classified in one of the following error types: nonword, semantic, formal, mixed, unrelated, omission and other.

Word production across different modalities was assessed using the PALPA 53 subtests (naming, repetition and oral reading). To determine if AM's nonwords were generated with reference to the target phonology, we evaluated the quality (i.e., the phonological relatedness between the target and nonword error) of his nonwords in production tasks using the Phonological Overlap Index (POI), with a resultant score ranging from 0 to 1 (Bose 2013, Folk *et al*. 2002, Schwartz *et al*. 2004). We used POI in preference of other similar measures (Robson *et al*. 2003, Olson *et al*. 2007), as it takes into account both target and error length, and averts the challenge that a longer error has a greater likelihood of including a target phoneme by chance.

POI =( Number  of  shared  phonemes  in  any  position  in  target  and  error )×2/ Phonemic  length  of  target + phonemic  length  of  error

#### Phonological versus semantic cueing experiment

A four‐session computerized picture naming experiment was developed using the 175‐item PNT, which has been successfully used by other studies in our laboratory (Bose and Buchanan 2007, Meteyard and Bose 2018). These four testing sessions manipulated cue condition (semantic versus phonological) and cue type (valid versus control). The sessions were blocked by cue condition (Meteyard and Bose 2018 provide a full description of the experimental manipulation). For each cue condition, testing was conducted over two sessions such that the items that were preceded by valid cues in one session were preceded by control cues in the other, and vice versa. For the phonological cueing sessions, the auditory cue was either the first sound of the name of the picture (e.g., /ball/ → ‘b’) in the valid condition or 1 kHz pure tone in the control condition. For semantic cueing, the valid auditory cue was a semantically associated word (e.g., /candle/ → ‘wick’) or a semantically non‐associated word in the control condition (e.g., /candle/ → ‘chop’). For the semantically associated control, we used the first associates of the target items in the University of South Florida Word Association Norms (Nelson *et al*. 1998). A trial consisted of the presentation of the recorded auditory cue, followed by 750 ms of silence and then the target picture, which remained on the computer screen until a response was made or a maximum of 10 s. The presentation of stimuli was randomized within sessions, with cue condition and type counterbalanced across sessions. Thus, a total of 700 picture naming trials [175 items × 2 cue conditions (semantic and phonological) × 2 cue types (control and valid)] were performed. All sessions were recorded with a high‐quality digital audio recorder.

#### Analysis of background testing battery to profile AM's deficits

We implemented Crawford and colleagues’ method for comparing single cases to a small control group (one‐tailed significance testing, Crawford and Howell 1998; effect sizes and confidence interval, Crawford and Garthwaite 2002, Crawford *et al*. 2010). These methods are preferable to converting to *z*‐scores as it does not treat the control samples as populations, but takes into account small sample sizes. We tested whether AM's score on each task was significantly lower than the control sample, and also compared AM's performance across tasks (e.g., across modality effects). The PNT error responses were used to calculate the error profile for which the error proportion was calculated by dividing the number of each error type by the total number of errors. The POI values for nonwords produced during picture naming (PNT), word repetition (PALPA 7) and nonword repetition (PALPA 8) were calculated. A higher POI would be indicative of a closer reference to the target phonology. We compared accuracy of each cueing condition (i.e., separately for phonological and semantic) for valid and control cues using a nonparametric related sample test (i.e., McNemar Change Test).

### Effect of PCA therapy on naming abilities

#### Re‐learning (trained) and response generalization (untrained) stimuli

The stimuli for the therapy consisted of 30 training and 30 generalization items, which were nouns from a variety of semantic categories. These 60 items were divided into six equal sets of 10 items each (three treatment lists, T1–T3; and three generalization lists, G1–G3). Across the six lists, items were matched on number of phonemes, syllable length, word frequency, number of phonological neighbours, imageability, concreteness and familiarity. AM's items were chosen from a set of 98 normed coloured photographs (previously used in research; Bose 2013) in consultation with him and his wife to keep them ecologically valid. In the initial selection, AM and his wife chose 75 items, knowing that only 30 of these would be trained and 30 would remain untrained, but used to measure generalization. A higher number of items was requested in order to provide sufficient range to match the items on their lexical variables. Allocation of the items to training or generalization lists was random. Appendix A provides the stimuli list.

#### Design for the therapy study and probes for naming performance

We used a single‐subject experimental design (i.e., a multiple‐probe design across behaviours), in which probe data were collected for all target behaviours across all phases of the treatment (Horner and Baer 1978). For better comparison between the present investigation and previous studies using PCA (e.g., Bose 2013, Leonard *et al*. 2008), the sequence of treatment item lists, probe frequency and administration were identical. The sequence of the design was as follows. First, before introducing the therapy of T1, six word lists were base rated three times to establish baseline naming accuracy. The pictures were shown one at a time, in random order, and AM was asked to name each. The first complete response was scored for accuracy. A response time of 10 s was imposed and no feedback was provided by the examiner. Percentage of correct names was calculated for each list.

During the therapy phase, teaching and/or learning criteria were implemented for each treatment list. The criterion for progressing to a subsequent list was 80% correct over two consecutive sessions or a maximum of 10 therapy sessions, whichever occurred first. The therapy and probes were administered by the same person, and each session was audio and video recorded for scoring verification and reliability. Therapy commenced with T1, presented as the first behaviour. Probes identical to those in the baseline phase were conducted throughout the therapy phases for all six lists. Therapy probes were collected during every session, but maintenance and baseline probes were conducted intermittently to minimize the effects of repeated exposure. For example, while T1 was used in the therapy phase, baselines were drawn from the five remaining word lists periodically (T2–T3 and G1–G3). After reaching the criterion for the first behaviour (T1), therapy was then applied to the second behaviour (T2). While T2 was in the therapy phase, probes continued to be collected for T1 as a means of monitoring maintenance, and for T3 and G1–G3 as baseline performance. Upon reaching the criterion for the second behaviour, therapy was discontinued with T2 and started with T3. While continuing therapy with T3, maintenance probing continued for T1 and T2. Finally, after reaching the criterion on T3, the therapy was discontinued.

#### Details on the PCA therapy procedure

Therapy sessions occurred thrice weekly for approximately 50–60 min at AM's home. For each training list, 10 therapy sessions were conducted, amounting to a total of 30 sessions. Presentation of therapy items was randomized for each session. The protocol for PCA therapy was identical to Leonard *et al*. (2008).

Each target picture was presented in the centre of a whiteboard and AM was asked to name it. This was the first naming attempt (i.e., naming before presentation of any cues). Regardless of the ability to name the picture, AM was asked to identify five phonological components related to the target word—rhyme, first sound, first sound associate (i.e., another word beginning with the same sound), final sound and number of syllables. If AM could not provide a spontaneous response to the rhyme and the first sound associate, he was given a list of written words on a card to choose from. If AM could not spontaneously provide a response for the first sound, final sound or number of syllables, a card with the correct response was provided on the white board. These choices were presented both visually (on a card), and verbally (by the examiner). Once a response was provided or chosen, the examiner wrote this response on the whiteboard. At no point during this process was the target word provided in written format. Once all of the components had been generated, AM was asked to name the target picture again; however, this time positive feedback was given if the response was correct (‘Yes, that's right. It's a _____’). This was the second naming attempt (i.e., naming after presentation of the cues). A correct response was given if AM was incorrect, which he was then encouraged to repeat. All phonological components of the target were reviewed by the examiner, regardless of whether or not AM was able to provide the correct responses. Following this review, AM was asked once more to name the target, followed by the positive or corrective feedback. This was deemed the third naming attempt (i.e., naming after reviewing the cues). During therapy for a list (i.e., 10 items), AM completed each item once within each session. Therefore, each word was exposed 10 times as there were 10 sessions. Each word had three opportunities to be named, first, second and third naming attempt, and the examiner was able to provide the correct response of each target *only two* times, regardless of whether or not AM had been able to name the target without support. On average, the completion of the PCA steps for each word took 3–4 min.

#### Analysis of re‐learning (trained) and generalization (untrained) of naming responses

**Figure 1:**
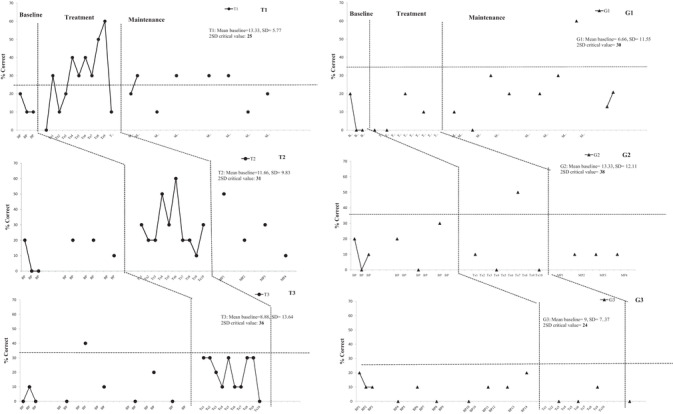
Naming accuracy on the treated word lists (T1–T3) and generalization word lists (G1–G3) across the baseline, therapy and maintenance phases for AM. The notation on the *x*‐axis (baseline probes, BP; therapy probes, TP, and maintenance probes, MP) indicates the number and type of probes. The vertical lines indicate different phases of the study; the horizontal dotted lines indicate the 2 SD critical cut‐off for the therapy performance to be significant.

The dependent measure for evaluating change during therapy was the percentage of correctly named pictures in probes. Responses were considered correct if they were phonologically accurate productions of the target word, occurring within 10 s of stimulus presentation. A multiple‐baseline format graph (figure 1) was used to present and analyze AM's performance (percentage accuracy) in the baseline, therapy and maintenance phases for both training and generalization items. The current literature offers several ways of analysing single‐subject therapy data (e.g., Howard *et al*. 2015, Nourbakhsh and Ottenbacher 1994, Scruggs and Mastropieri 2013). We used two statistical analyses to quantify the change during therapy: the 2 SD (standard deviation) band method (Nourbakhsh and Ottenbacher 1994) and effect sizes (Busk and Serlin 1992, Kromrey and Foster‐Johnson 1996). We used these methods to facilitate the comparison of AM's data with those of P9 and FF (Bose 2013, Leonard *et al*. 2008).

## Results 1

### Identifying impaired and preserved processes to localize AM's deficit

Table 2 presents AM's performance on the battery of background testing along with the results of statistical analysis using Crawford and colleagues’ methods (Crawford *et al*. 2010, Crawford and Howell 1998). AM's scores on conceptual and lexico‐semantic processing tasks (i.e., PPT, PALPA 47 and 48) did not indicate impairment when compared with normative data. However, he did show a lower score in auditory synonym judgement, which may be attributable to his difficulties in auditory comprehension or input phonological processing, or some difficulties in the lexico‐semantic processing. AM demonstrated severe word production deficits. On picture naming tasks (PNT and PALPA 53), AM performed significantly worse than normal speakers, with large effect sizes. AM also showed significant impairment on tests of oral reading and repetition on the PALPA 53; however, effect sizes were much smaller when compared with picture naming. Comparison of AM's performance on naming, oral reading and repetition using the same PALPA 53 items showed significant differences amongst the tasks (*χ* ^2^ = 30.62, *p* < 0.00), with naming being most impaired followed by repetition and then oral reading (naming versus repetition, *χ* ^2^ = 11.31, *p* < 0.00; naming versus reading, *χ* ^2^ = 29.46, *p* < 0.00; repetition versus reading, *χ* ^2^ = 5.59, *p* = 0.02).

**Table 2:** AM's performance on various linguistic and neuropsychological tests

		AM's performance		Control sample			Significance test^a^		Estimated effect size (*z*_cc_)^b^		Estimated% of the control population obtaining a lower score than the case	
	Total number of items	Raw score	%	*n*	Mean	SD	*t*	*p*	Point	95% CI	Point	95% CI
*Input and output phonology* c												
PALPA 2: Real word minimal pair discrimination	72	45	63									
Same	36	28	78	24	35.54	0.78	–9.47	0.00	–9.66	–12.46 to –6.86	0	0 to 0
Different	36	17	47	24	34.83	2.58	–6.77	0.00	–6.91	–8.92 to –4.88	0	0 to 0
PALPA 4: Minimal pair requiring picture selection	40	31	78	25	39	1.7	–4.61	0.00	–4.71	–6.08 to –3.32	0	0 to 0.02
PALPA 5: Auditory lexical decision												
Word stimuli	80	70	88	21	79.43	1.37	–6.72	0.00	–6.88	–9.04 to –4.72	0	0 to 0
Nonword stimuli	80	54	68	21	76	4.27	–5.03	0.00	–5.15	–6.79 to –3.51	0	0 to 0
PALPA 9: Word repetition (Imageability × Frequency)	80	25	31	21	78.81	2.71	–21.2	0.00	–21.3	–27.8 to –14.7	0.00	0 to 0
High imageability	40	17	43	21	39.33	1.53	–13.6	0.00	–13.9	–18.2 to –9.6	0.00	0 to 0
Low imageability	40	8	20	21	39.48	1.18	–30.2	0.00	–30.9	–40.4 to –21.3	0.00	0 to 0
High frequency	40	14	35	21	39.62	1.2	–23.7	0.00	–24.3	–31.7 to –16.8	0.00	0 to 0
Low frequency	40	11	28	21	39.19	1.51	–18.9	0.00	–19.3	–25.2 to –13.7	0.00	0 to 0
PALPA 7: Word repetition	24	7	29		Unavailable							
One syllable	8	2	25									
Two syllable	8	2	25									
Three syllable	8	3	38									
PALPA 8: Nonword repetition	30	2	7		Unavailable							
One syllable	10	1	10									
Two syllable	10	0	0									
Three syllable	10	1	10									
*Auditory monitoring* d	40	39	98									
Monitoring of one's own naming response on the PALPA 53 items on a different day												
*Conceptual and lexico‐semantic processing* c												
Pyramid and Palm Tree (three‐picture version)	52	49	94	70	89.84	7.75	0.56	0.29	0.56	0.31 to 0.81	71.2	62.1 to 79.2
PALPA 47: Spoken word–picture matching	40	40	100	31	39.29	1.07	0.65	0.26	0.66	0.26 to 1.05	74.1	60.6 to 85.3
PALPA 48: Written word–picture matching	40	39	98	32	39.47	1.01	–0.46	0.33	–0.46	–0.83 to –0.09	32.5	20.4 to 46.14
PALPA 49: Auditory synonym judgments	60	32	53	5	Unavailable							
High imageability	30	19	63	5								
Low imageability	30	13	43	5								
*Word production across modalities* c												
53: Picture naming	40	11	28	29	39.8	0.35	–80.9	0.00	–82.3	–103.7 to –60.8	0	0 to 0
53: Oral reading	40	35	88	28	39.96	0.19	–25.6	0.00	–26.1	–33.0 to –19.1	0	0 to 0
53: Repetition	40	26	65	28	39.79	0.83	–16.4	0.00	–16.6	–21.0 to –12.2	0	0 to 0
*Philadelphia Naming Test* c												
Accuracy	175	35	20	18	164.28	5.65	–24.9	0.00	–25.54	–34.0 to –17.0	0	0 to 0
Proportion of nonwords		54	0.39									

Notes: ^a^One‐tailed significance testing based on Crawford and Howell (1998).

^b^Effect sizes and confidence interval based on Crawford and Garthwaite (2002) and Crawford *et al*. (2010). Control data for the PALPA subtests and PPT were drawn from the test manual, whereas control data for the PNT were obtained from on 18 monolingual English‐speaking British participants.

^c^Background testing performed within a 2‐month period before therapy.

^d^Auditory monitoring was performed 1 year before the start of therapy.

The grey shaded areas indicate a significant difference.

AM showed impaired word and nonword repetition, with nonwords being more severely impaired than real words. The PALPA 9 tested frequency and imageability effects in word repetition, on which AM showed neither frequency (*χ* ^2^ = 0.46, *p* = 0.23) nor imageability effects (*χ* ^2^ = 2.56, *p* = 0.1). AM also showed impaired performance on input phonological processes. Although, we did not explore auditory monitoring in detail, AM showed almost ceiling performance on a delayed auditory monitoring task.

Nonword was the most dominant error type in all production tasks: PNT (54 nonwords, proportion about 0.39); PALPA 7 word repetition (15 nonwords, proportion about 0.88); and PALPA 8 nonword repetition (23 nonwords, proportion about 0.82). The mean POIs for nonwords on the PNT, word repetition and nonword repetition were 0.19 (SD = 0.15), 0.50 (SD = 0.14) and 0.57 (SD = 0.17) respectively. The mean POIs on the repetition tasks were significantly higher than that on picture naming.

Analysis of AM's accuracy scores from the two cueing conditions showed a significant increase in naming accuracy following phonological cueing (phonologically valid 34/175, phonologically control 18/175; *χ* ^2^ = 5.78, *p* = 0.01), whereas semantic cueing did not show this effect (semantically valid 26/175; semantically control 23/175; *χ* ^2^ = 0.21, *p* = 0.64). Table B1 in Appendix B shows a distribution of error types across the different cueing conditions illustrating comparable distribution across the cueing types.

### Re‐learning (trained) and generalization (untrained) of naming responses following PCA therapy

Figure 1 illustrates the multiple probe data representing the percentage of correct names for treated and untreated words from the probes collected during the baseline, therapy and maintenance phases. The set of graphs in the left and right panels represent the data for treated lists T1–T3 and untreated lists G1–G3 respectively, ordered from top to bottom in accordance with the sequence in which therapy was administered. As seen, AM demonstrated low baseline scores across all sets. The mean baseline scores for T1 was 13% (SD = 5.8), T2 was 12% (SD = 9.8), T3 was 9% (SD = 13.6), G1 was 7% (SD = 11.5), G2 was 13% (SD = 12.1) and G3 was 9% (SD = 7.4).

With PCA therapy, the naming responses improved for T1 and T2; albeit to different degrees, whilst T3 did not show any improvement. During the therapy phase, naming accuracy in T1 increased from 0% to 60% (mean = 32%, SD = 16.2), in T2 from 10% to 60% (mean = 29%, SD = 15.2) and in T3 there was change from 0% to 30% (mean = 20%, SD = 11.6). During the maintenance phase, the mean naming accuracy for T1 and T2 was 22.5% (SD = 8.9) and 24.5% (SD = 16.9) respectively. Note that for lists T1 and T2, AM showed marked variability in his performance during therapy phases. Most notably, he reached the highest score of 60% for these two lists, but therapy gains tended to revert back to pre‐therapy accuracy levels towards the end of the therapy phases, as well as when therapy was discontinued (i.e., maintenance phase). The generalization lists G1–G3 showed a mean performance of 10% (SD = 13), 15% (SD = 23.9) and 3.3% (SD = 5.8) during the therapy phases.

Using the 2 SD band analysis method, therapy performance is considered improved if at least two consecutive data points in therapy exceeded the 2 SD critical cut‐off value. For T1–T3 the critical cut‐offs were 25, 31 and 36 respectively, represented by a horizontal dotted line in figure 1. Based on the 2 SD criteria, only T1 and T2 showed improvement with therapy. This finding corroborates the results of the effect size analyses. The effect sizes for lists T1–T3 were 3.2, 1.8 and 0.8 respectively. Based on Busk and Serlin (1992), the effect sizes for T1 and T2 would be large and medium respectively. The performance on generalization lists G1–G3 showed considerable variability; however, these changes were not sufficiently consistent to qualify as improvement based on the visual or statistical analyses (i.e., no consecutive data points beyond 2 SD critical cut‐off, and effect sizes for lists G1–G3 were 0.3, 0.2 and –0.8 respectively).

In summary, results from the PCA therapy were: (1) a benefit from PCA for two of AM's treated word lists during the re‐learning phase, but these gains were not maintained; and (2) no demonstrable generalization to the untreated items.

## Discussion 1

Based on AM's performance on a wide range of tasks, we propose that his naming impairments arise primarily from impairment in the connection between semantic to phonological systems, that is, a deficit in accessing the phonological word forms from the semantic system. This deficit is also referred to as a deficit in lexical access (Howard and Gatehouse 2006, Robson *et al*. 1998, Van Hees *et al*. 2013), and has previously been noted in other cases of jargon aphasia (e.g., Bose and Buchanan 2007, Hickin *et al*. 2002, Hillis *et al*. 1999).

AM demonstrated: *relatively* preserved conceptual and lexico‐semantic processing abilities; severe deficits in word production across all modalities with nonword being the dominant error; picture naming being significantly worse than repetition and oral reading; and lower phonological relatedness between the target and nonwords during picture naming than that of repetition tasks. AM exhibited relatively preserved delayed auditory monitoring abilities. He evidenced increased naming accuracy with phonological cueing. AM's profile is consistent with the profile of individuals having a deficit in the link from semantics to phonology (e.g., Howard and Gatehouse 2006, Van Hees *et al*. 2013). When provided with a phonological model of the target, as in reading (visual model) and repetition (acoustic model), the phonological relatedness of AM's nonwords was higher compared with that in naming, indicating improved access to the phonological forms without access through semantics. Increased accuracy to phonological cues have been argued to demonstrate the existence of a mechanism for overcoming the connection problem from semantics to phonology (Bose 2013, Hickin *et al*. 2002, Howard and Gatehouse 2006, Lambon‐Ralph *et al*. 2000, Miceli *et al*. 1996, Van Hees *et al*. 2013). Therefore, an increase in phonological activation either by providing the phonological form, through repetition and oral reading, or by means of phonological cueing improved AM's access to these phonological forms.

Improved naming ability on two of AM's treated word lists following PCA corroborates previous research on the usefulness of phonological therapy approaches in jargon aphasia in particular (Bose 2013, Robson *et al*. 1998), and in aphasia in general (e.g., Hickin *et al*. 2002, Leonard *et al*. 2008, Van Hees *et al*. 2013). The use of a single‐subject design across behaviours allowed us to demonstrate the therapy gains twice (i.e., T1 and T2). However, AM did not show a benefit of PCA on his third treated list, and the gains he achieved during therapy reverted back to pre‐therapy levels towards the end of the therapy phases and after therapy was discontinued. These findings of short‐term gains without long‐term maintenance are not surprising in consideration of the established intractability of jargon aphasia (Marshall 2006, 2018).

Of the three existing therapy studies on naming difficulties in jargon aphasia, the study using semantic therapy did not show a positive outcome (Marshall *et al*. 1998); however, those using phonological therapies did show *some* positive results in re‐learning (Bose 2013, Robson *et al*. 1998). Several factors could have contributed to the relative success of the phonological therapies. Relatively preserved repetition and oral reading skills could support phonological access. Research has reported a significant positive correlation between oral reading and the outcome of phonological therapy (Bose 2013, Hickin *et al*. 2002, Leonard *et al*. 2008). Phonological cueing acts (in part) to boost phonological activation of the target phonemes and could thereby facilitate the mapping between semantics and phonology. We propose that the phonological cues provided in PCA facilitated AM to access phonology, thus enabling him to successfully name several of the trained items from the first two lists. Participants GF in Robson *et al*. (1998) and FF in Bose (2013), who showed improvement in picture naming following phonological therapy, were postulated as having difficulty in accessing phonology from semantics. We suggest that this may provide evidence of a similar underlying deficit in mapping between semantics and phonology amongst AM, FF and GF. The use of phonological therapy to overcome such difficulties could potentially strengthen the case for using phonological therapy for the amelioration of naming difficulties in jargon aphasia, at least in the re‐learning phases. However, we need to be mindful that variability in performance and the magnitude of benefit remains limited in these populations (Brady *et al*. 2016). Moreover, jargon production could be the result of other underlying deficits within the word production system, such as phonological distortion of the target and/or error during phonological encoding (e.g., Kertesz and Benson 1970) or difficulty in accessing the phonological output lexicon (Kohn *et al*. 1996). In addition, the number of published studies is as yet limited, and systematic research comparing effectiveness of semantic and phonological approaches for this group is non‐existent. Future research exploring the linkage between the underlying source of deficit for jargon aphasia and type of therapy applied could shed a better light on the existing unresolved issues in this field.

There are reports of individuals with jargon aphasia. For example, P9 in Leonard *et al*. (2008), who closely resembled AM and FF in her profile, and did not benefit from PCA. The second analysis investigates the possible factors that might have contributed to disparate outcomes to PCA despite similar therapy, dosage and clinical profiles amongst individuals with jargon aphasia.

Full consideration must be given to the possible causes for AM's drop in naming performance towards the end of the therapy phase and the lack of maintenance. Instantiating long‐lasting change in behaviours following neurological damage is an oft‐reported challenge in the field of rehabilitation (e.g., Martin *et al*. 2006). It is possible that the mechanism through which AM made use of the phonological cues could explain the inability to retain the benefits of phonological cueing. The cues provided during PCA encourage a participant to engage actively in feature generation and processing of the cues. Active manipulation of cues and deeper processing give rise to enhanced and strengthened semantic and phonological associations, and should enable long‐lasting therapy benefits (Leonard *et al*. 2008). However, researchers have argued that if the gain in therapy is by way of priming mechanisms, then the effects are usually reduced and short lived (Howard *et al*. 2006, but see Cave 1997for long‐lasting priming effects in healthy adults).

AM did not show any improvement in naming the untreated items. This lack of generalization is not surprising in light of a review of key anomia studies reported by Best *et al*. (2013), who found that only one‐quarter of adults with aphasia demonstrate generalization in word production, whilst studies on jargon aphasia using phonological therapy have reported limited or inconsistent generalization. For example, FF and P9 showed no generalization to PNT items (Bose 2013, Leonard *et al*. 2008), whilst GF did make some gains on untreated items (Robson *et al*. 1998). It has been suggested that the mechanism employed in phonological therapy requires strengthening of links between the semantic system and phonological output lexicon (Nickels and Best 1996), and that this mapping is item specific (Howard 2000, Hickin *et al*. 2002, Miceli *et al*. 1996). If AM's predicted difficulty was with the mapping between semantics and phonology, then it is possible that the mechanism through which he benefitted in the naming task was in fact item specific. However, recent literature has identified that phonological cues interact differentially with certain lexical and image properties. For example, Meteyard and Bose (2018) have shown that phonological cues interact with image properties rather than lexical variables; whilst Conroy *et al*. (2012) have shown that highly imageable items needed less cueing and were named more accurately. It is possible that many of the findings in the literature of cueing in aphasia could be driven by the interaction of cues and the psycholinguistic properties of the words. This remains a rich area for future research to explore.

### Retrospective analysis of three cases of jargon aphasia

The varying therapy outcomes amongst jargon aphasia participants could be accounted for by a number of factors: therapy type (semantic versus phonological), severity of naming deficits, differences in linguistic abilities, dosage of therapy and/or ability to engage with therapy/cues. Since therapy success has been limited with individuals with jargon aphasia, an important clinical goal would be to find possible determiners of success for therapies.

We have been fortunate in having access to the assessment and therapy data (generation and use of the phonological cues) for three individuals with jargon aphasia, one from the current study (AM) and from two previously reported in the literature: P9 (Leonard *et al*. 2008), and FF (Bose 2013), who performed differentially in PCA therapy. All three participants showed similar clinical manifestations and underwent a similar dosage (number of sessions) of PCA to improve their naming. Despite these similarities, they responded differently to the therapy: P9 did not show any positive gains; FF showed improved naming and maintained some of the gains; and AM showed some improvement in naming, but did not maintain his gains. This study provides a retrospective analysis of these three individuals in an attempt to determine the prognostic indicators for successful candidacy in using phonological therapies in jargon aphasia.

First, we examined the participants’ differences and similarities in performances on various linguistic tasks to decipher if these could explain their differential responses to PCA. Second, we investigated whether therapy outcomes depended on the participant's ability to engage in the therapy (i.e., ability to generate and use the cues). PCA therapy requires identification and generation of five phonological components—a rhyming word, first sound, first sound associate, final sound and number of syllables—for each target word. A comparison of performances in generating and using the phonological cues would be an indication of how individuals engaged in the therapy, and if this can impact re‐learning and maintenance of naming responses. Third, participants had three opportunities to name each word during therapy: first (i.e., naming before presentation of cues), second (i.e., naming after presentation of cues) and third (i.e., naming after reviewing cues). A comparison of performance across the three naming attempts could shed a light into an individual's ability to retain and maintain the cues, which could determine whether they were able to use the phonological cues in the short versus the long term. Specific research questions were:

- Did differences or similarities in the underlying linguistic skills of P9, FF and AM contribute to their differential performance in therapy?
- Did P9, FF and AM show differential abilities to generate and use the phonological cues as measured by their feature generation in PCA therapy?
- Did P9, FF and AM show differential performances amongst the three naming attempts during the PCA therapy?

## Methods 2

### Participants and therapy outcome

Table 3 provides demographic information on P9, FF and AM, their BDAE results, narrative samples from the Cinderella story, and their responses to PCA therapy. The assessment results on the background tasks for P9 and FF along with data on generation and use of the phonological cues during therapy were made available to us by the respective authors; AM's data were collected by us. All participants were classified as having severe fluent aphasia, with poor auditory comprehension, poor repetition and severe word‐finding difficulty, along with the production of nonwords being the most dominant error type in their picture naming. Using a single‐subject multiple‐probe design across behaviours, PCA therapy was employed with all three participants to ameliorate their naming difficulties, with all participants receiving similar frequency (thrice weekly) and dosage (about a total of 30 h) (table 3).

**Table 3:** Clinical profiles of the three participants (P9, FF and AM) and their response to phonological component analysis (PCA) therapy

	P9	FF	AM
*Demographics*			
Age (years)/gender	72/Female	77/Male	85/Male
Aetiology	Single LCVA^a^	Single LCVA^a^	Two LCVAs^a^
Lesion	Left temporo‐parietal	Left temporo‐parietal	Left temporo‐parietal
Years post‐onset	1.5	4	15
Language background	Monolingual English speaker	Monolingual English speaker	Monolingual English speaker
*BDAE* b *profile*			
Aphasia type	Wernicke's	Wernicke's	Conduction
Aphasia severity	1	1.5	2
Word comprehension^c^	10th	40th	60th
Commands^c^	20th	20th	70th
Complex ideational material^c^	20th	20th	10th
Word repetition^c^	20th	40th	10th
Sentence repetition^c^	20th	40th	60th
Boston naming (short form)^c^	10th	30th	30th
Proportion of nonwords in PNT^d^	High, 0.46	High, 0.44	High, 0.39
Sample from the Cinderella Narrative	..new was gonna carry the new /p:p/ /pu:p/ the /eɪkən/ for their theory to meet their their /fɪlt/, and guess they were all trying to get the, the the that fact their feet would fit in their in their shoe in their right place and … nobody had…found that…yet, no one they were all had to be /krɪsi:/ feet, an‐ they …weren't people the nice they didn't have the little baby feet like …um uh the lil girl /bu:bəl/ /bu:bəlfᴐt/ had…	and looks like the /lʌɡi:bɜrɡəz/. It says oh we're gonna to pick a /lɪɡi:bɜrɡə/ that we want to get our /lɪɡi:bɜrɡəz/. And so they, the..the king say or the so the men the uh the /pɪɡi:bɜrɡə/ say ah well here's the /bɪɡi:ɡɜrɡə/ and /bləʊblӕ:/ and all the rest of it and so they…they….they have a big big thing. And so the queen ago or the old /ɡɪɡi:bɜrɡəz/ they all /ʃodəʊ/ /bɛtӕ/ /sɪki:/ /pɛtəɡi:bɜrɡə/…	She small girl…/mændɪŋ/…favourite man /sowɔ/ him….and this is, this is her /mɪŋ/ sisters….and there's three girl comes along, and their /mɛn/ on a /drɪps/…and they nasty senior…nasty /sɪŋdʒiʒa/, nasty girls….princess is outside the stage…an /deɪta/ awaiting for him…she n‐out the window and she /sɔmənʃiæ/…something pink made her…and the manager…says /sutɪŋ/ on stage…many girls waiting for the three sisters…she's reading a book and she's looking at something…/manæzəha/
*PCA therapy*			
Total hours	30	33	30
Frequency	Thrice a week	Thrice a week	Thrice a week
Duration of each session (min)	50–60	50–60	50–60
Number of treated items	30	30	30
Effect sizes	List 1: n.a.; List 2: n.a.^e^	List 1: 2.0; List 2: 2.8; List 3: 2.7	T1: 3.2; T2: 1.8; T3: 0.8
Generalization	No generalization on pre‐ and post‐PNT naming	No generalization on pre‐ and post‐PNT naming	No generalization to untreated items
Response to therapy	No positive gains	Positive gains and maintained	Some positive gains but not maintained

Notes: ^a^Left cerebrovascular accident.

^b^Boston Diagnostic Aphasia Examination (Goodglass *et al*. 2001).

^c^Percentile score.

^d^Philadelphia Naming Test (Roach *et al*. 1996).

^e^P9 baseline performance on probes was at the floor level, thus precluding effect size calculation.

P9 did not demonstrated a positive therapy effect (Leonard *et al*. 2008: 941, fig. 11). FF showed significant improvement in his ability to name pictures on all three lists (Bose 2013: 588, fig. 1), whilst AM showed significant improvement on only the first two treated lists. FF maintained his therapy gains, whereas AM did not. Table 3 provides the number of items treated for each participant, effect sizes and generalization to other naming task. Notably, none of the participants showed generalization to these other naming tasks (i.e., no improvement from pre‐ to post‐therapy PNT performance, for FF and P9, and no improvement for generalization items for AM).

### Tasks to identify linguistic differences and similarities amongst P9, FF and AM

**Figure 2:**
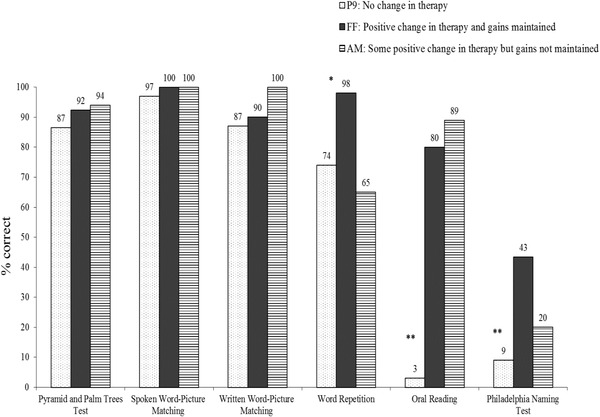
P9, FF and AM's performance (percentage correct) across various linguistic tasks. ^**^Significant difference at alpha set at 0.01; ^*^significant difference at alpha set at 0.05.

Data collected on several linguistic tasks tapping into conceptual, semantic and phonological processes from the previous studies (Bose 2013, Leonard *et al*. 2008) along with the current investigation, were used. Table 3 and Figure 2 provide the word lists, as well as the tasks and tests.

### Phonological feature generation during PCA

Data for P9 and FF were provided to us by the studies’ respective authors. Data on each participant's ability to generate the five phonological features—rhyming words, first sound, first sound associate, final sound, and number of syllables—for each word in every session were used. These were then averaged for each word list. Mean scores for each feature on each list were converted to percentages respective to each feature (e.g., percentage of rhymes generated). Table 4 presents the feature summary for the three participants.

**Table 4:** Summary of phonological feature generation (percentage of correctly generated feature) for the three participants during PCA therapy for treated words

		Rhymes	First sound	First sound associate	Last sound	Syllable	Average
P9	List 1	0	2.7	8	12	74	19
	List 2	3	15	15	22	77	26
	Mean	**2**	**9**	**12**	**17**	**76**	**23**
FF	List 1	31	42	10	44	60	37
	List 2	60	38	16	73	80	53
	List 3	48	38	21	65	72	49
	Mean	**46**	**39**	**16**	**61**	**71**	**46**
AM	T1	0	1	61	0	41	21
	T2	0	1	69	0	42	22
	T3	1	2	70	1	35	22
	Mean	**0**	**1**	**67**	**0**	**39**	**22**

### Performance on three naming attempts for each word during PCA

Naming accuracy was tracked for every word on each list on all the three naming attempts: first (i.e., naming before presentation of cues), second (i.e., naming after presentation of cues) and third (i.e., naming after reviewing cues). This score was converted into mean percentage correct for each treated list (table 5).

**Table 5:** Naming accuracy (percentage correct) across the three naming attempts (first, second and third) for the three participants during PCA therapy

		First naming attempt	Second naming attempt	Third naming attempt
P9	List 1 (10 items × 15 sessions)	14	13	19
	List 2 (10 items × 15 sessions)	34	29	11
	Mean	**24**	**21**	**15**
FF	List 1 (10 items × 12 sessions)	64	78	93
	List 2 (10 items × 9 sessions)	82	86	93
	List 3 (10 items × 12 sessions)	68	83	95
	Mean	**71**	**82**	**94**
AM	T1 (10 items × 10 sessions)	34	40	32
	T2 (10 items × 10 sessions)	21	47	33
	T3 (10 items × 10 sessions)	21	50	44
	Mean	**25**	**46**	**36**

## Results 2

### Linguistic differences and similarities amongst P9, FF and AM

Figure 2 shows the performance (percentage correct) across various semantic and phonological tasks for all three participants. The raw scores and *p*‐values on the semantics tasks were: PPT (P9 45/52, FF 48/52, AM 49/52; *χ* ^2^ = 2.04, *p* = 0.36); spoken word–picture matching (P9 38/39, FF 40/40, AM 40/40; *χ* ^2^ = 0.25, *p* = 0.88); and written word–picture matching (P9 34/39, FF 36/40, AM 39/40; *χ* ^2^ = 1.63, *p* = 0.44). Owing to an administration error for testing with P9, only 39 items were presented on the PALPA 47, 48 and 53. Analyses showed comparable performances amongst the three participants on tasks of conceptual and lexical semantics. The raw scores and *p*‐values on the PALPA 53 subtests were: word repetition (P9 29/39, FF 39/40, AM 26/40; *χ* ^2^ = 7.11, *p* = 0.03; P9 versus AM, *χ* ^2^ = 0.36, *p* = 0.43; P9 versus FF, *χ* ^2^ = 6.99, *p* = 0.01; FF versus AM, *χ* ^2^ = 11.81, *p* < 0.00) and oral reading (P9 1/39, FF 32/40, AM 35/40; *χ* ^2^ = 66.69, *p* < 0.00; P9 versus AM, *χ* ^2^ = 54.06, *p* < 0.00; P9 versus FF, *χ* ^2^ = 45.55, *p* < 0.00; FF versus AM, *χ* ^2^ = 0.37, *p* = 0.54). Naming accuracy on the PNT showed a significant difference amongst participants (P9 16/175, FF 76/175, AM 35/175; *χ* ^2^ = 58.60, *p* < 0.00). The following pattern of results emerged: (1) differences in the performance on lexico‐semantic tasks and word repetition skills did not discriminate between the individuals who responded successfully to PCA and those who did not; and (2) FF and AM both benefitted from PCA, albeit to different degrees, and showed better oral reading abilities compared with P9.

### Phonological feature generation during PCA

Table 4 presents the feature generation summary for the three participants. The mean percentage of phonological features generated by P9, FF and AM was 23%, 46% and 22% respectively, with FF producing significantly more features than P9 and AM (*χ* ^2^ = 17.46, *p* < 0.00). FF's ability to generate features improved from list 1 to 2, and remained similar on lists 2 and 3. Qualitative comparison of types of features provides a richer picture amongst the three participants. Both FF and AM demonstrated some phonological knowledge about the first sounds of words, albeit for AM this was restricted to the first sound associates. P9 performed poorly on both first sounds judgements, but was knowledgeable about syllable number judgements, similar to FF and AM. It may be that these sound segmentation abilities, especially first sound judgements, were associated with therapy effects. This would thereby suggest that in addition to an overall ability to generate features, one would need more specific skills, such as that of phonological segmentation, to benefit from this therapy.

### Performance on three naming attempts for each word during PCA

Table 5 shows the mean naming accuracy across treated word lists in all naming attempts for P9, FF and AM. There is a lack of change in P9's performance across the three naming attempts, whereas FF and AM show increased naming accuracy in the second naming attempt, suggesting utility of the benefits provided by the cues during therapy. FF, who had showed maintenance of PCA therapy gains, further improved his performance on the third naming attempt; however, AM did not show any further increase in naming accuracy from second to third attempt.

## Discussion 2

The first analyses revealed that all three of the participants had comparable performances in the semantic domain. These measures did not discriminate among the individuals with jargon aphasia who responded successfully to phonological therapy and those who did not. FF and AM both benefitted from PCA, albeit to different degrees, and showed better oral reading abilities when compared with P9. Note that FF also demonstrated better repetition abilities than P9 and AM, whose performances were comparable with each other. It is possible that the oral reading skills exhibited by FF and AM enabled them to use the phonological cues from therapy to help them name the picture stimuli; a claim that may be further supported by positive correlations between oral reading and success in naming therapy reported in previous research (e.g., Hickin *et al*. 2002, Leonard *et al*. 2008). In addition, the further preserved phonological abilities of FF in repetition may have contributed to their added successful therapy performance, which continued to the third attempt.

When evaluating therapy results, one might argue that differential treatment effects could simply be a function of the overall severity of naming deficit. P9, indeed, had the most severe naming impairment (PNT performance). Although overall naming severity does contribute to the therapy outcome in aphasia (Lambon‐Ralph *et al*. 2000), therapy studies involving individuals with jargon aphasia, do not reveal a straightforward relationship between the naming severity and therapy outcome. In Robson *et al*. (1998), GF improved following phonological therapy and had a more severe naming deficit than did CM, who did not improve with therapy. This report demonstrates that individuals with severe jargon aphasia can benefit from therapy, provided that some phonological abilities are relatively preserved and can be used in therapy, in this case, in oral reading for FF and AM, as well as repetition skills for FF.

The second analysis on the comparison of the participants’ feature generation skills during the PCA therapy illustrated that better performance on certain features are more closely linked to successful therapy performance than others. Overall, FF produced wider range of phonological features. Closer inspection of individual features generated by participants who improved in therapy, that is FF and AM, demonstrate both generated features related to the first sound, either generating the first sound and/or generating a word with the first sound. Syllable number judgment as a feature did not show differential performance amongst the three participants.

The comparison of the individuals’ performance on three naming attempts for each word allowed us to investigate the short‐ versus long‐term benefits of phonological cueing. Both FF and AM produced an increase in naming accuracy on the second naming attempt, illustrating the possible utilization of the benefits provided by cues. FF who showed maintenance of his therapy gains, further increased his performance on the third naming attempt. In contrast, AM did not show any further increase in naming performance from second to third attempt, with his performance deteriorating instead. These results suggest that AM was able to receive and use phonological cues in the short term, but unable to retain this benefit for a longer period. This highlights that even for individuals responding well to phonological therapy during the re‐learning phase, retention of these benefits for long‐term gains does not necessarily follow. In contrast, P9's performance across the three naming attempts did not change at all.

Withstanding the limitations of any retrospective analysis, the findings illustrate that the availability of oral reading abilities, active engagement in generation and use of the phonological cues, especially features related to sound segmentation, along with long‐term retention of the phonological cues, were necessary for achieving adequate re‐acquisition and successful maintenance of phonological therapy gains in jargon aphasia. Future research investigating pre‐therapy phonological segmentation abilities and its relation to phonological therapy outcomes would be a useful step to determine candidates who benefit from such approaches.

## General discussion

This research investigated: the locus of word production deficit in AM; the usefulness of a phonological therapy (PCA) to ameliorate his naming impairments; and possible factors that lead to differential therapy outcomes in jargon aphasia. The results revealed that AM had a deficit in the connection from semantics to phonology, which resulted in a difficulty in accessing the phonological word forms, when it was to be accessed via semantics. Previous research has reported on individuals with jargon aphasia, who have demonstrated a similar difficulty (e.g., Bose and Buchanan 2007, Hillis *et al*. 1999, Robson *et al*. 1998). The literature on aphasia therapy tends to suggest that if the naming impairment results from a connection impairment between semantics and phonology, then phonological cueing is an effective means of ameliorating this impairment (Hickin *et al*. 2002, Leonard *et al*. 2008, Van Hees *et al*. 2013).

We were able to test this assumption from the literature within a therapy context. This specifically allowed us to determine if strengthening signals to the phonological level would improve output in jargon aphasia, as predicted by Robson *et al*. (2003). The results from the PCA therapy with AM confirmed that phonological cueing therapy was indeed a useful approach to improve AM's naming responses during the re‐learning phase. However, gains were limited to two training lists with no generalization to untrained items. Importantly, AM was unable to maintain his therapy gains. Although, it is generally agreed that the treatment of individuals with jargon aphasia as a client group can present as challenging, these findings still demonstrate that phonological cues were helpful in improving naming responses, albeit variably, for AM at least in the re‐learning phase. We speculate that the cueing advantage demonstrated by AM was a result of short‐term priming effects rather than a long‐term retention and processing of cues. AM's success for feature generation was at about 22%, which could also be suggestive of his difficulty in actively generating and processing phonological cues. Individual features generated during PCA showed that he was successful in generating the first sound associate. This could also imply that he was receiving a benefit from the phonological information, but he was unable actively to generate or process all types of features.

From the evidence in the current study, to achieve long‐term gains, an individual would require an ability to engage and process cues actively and in depth. Comparison of the three individuals with jargon aphasia in the retrospective analysis further substantiates that relatively good performance on oral reading and/or repetition, along with the ability to actively process and engage with the cues, can result in long‐term benefits from phonological therapy.

## Conclusions

This study identified the locus of deficit in an individual with jargon aphasia, AM, to be at the level of connection from semantics to phonology, and provided further evidence of the usefulness of a phonological therapy for improving naming responses during the re‐learning phase of therapy. Therapy gains were not maintained in the long term and generalization to untreated picture names was not observed. AM's data were compared with two other individuals with jargon aphasia, who showed differing outcomes to the same phonological therapy, with the aim of determining possible indicators for therapy success. The observations suggest that to receive a benefit from phonological therapy, participants with jargon aphasia may require both good oral reading and/or repetition abilities and an ability to engage with phonological cues. Future research needs to address the many outstanding issues—the influence of cognitive and learning abilities on therapy gains, the relationship between the underlying deficit and mechanism of change in naming and nonwords, the use of a wider range of generalization tasks with clear motivations, and better means of documenting change in therapy—which would lead to a better understanding of the underlying deficit in jargon aphasia, and ultimately better therapy outcomes for their production difficulties. These lines of enquiry would also help to generate specific recommendations for the candidacy of different types of therapy (e.g., semantic versus phonological or amongst various types of phonological therapies) in jargon aphasia.

## Note

1 Leonard *et al*.’s study investigated PCA for improving naming in aphasia in general, whilst not focusing on jargon aphasia specifically. However, amongst their cohort of 10 participants, three did not show treatment effects. P9 was one of those three and presented with jargon aphasia.

## Appendix A Stimuli for therapy (training items T1–T3) and generalization (untrained items G1–G3) for phonological component analysis (PCA) therapy

T1 items: grape, frog, cherry, kettle, suit, train, lemon, snow, bed and elbow.
T2 items: belt, sheep, sweater, cheese, lamp, bread, desk, bus, banana and dentist.
T3 items: stool, iron, nurse, potato, lettuce, shirt, lips, sky, elephant and milk.
G1 items: lime, bear, tie, squirrel, leaf, fridge, cake, cookie, carrot and boot.
G2 items: star, table, toaster, chair, beach, tomato, dress, hat, ear and truck.
G3 items: chef, swan, onion, pear, fish, corn, organ, sailor, egg and waiter.

## Appendix B

**Table B1:** Profile of the naming responses from the phonological and semantic cueing experiments, and standard PNT naming

	Nonword		Formal		Semantic		Mixed		Unrelated		Other			
	*n*	%	*n*	%	*n*	%	*n*	%	*n*	%	*n*	%	Total errors *n*	Accuracy *n*
PNT (standard)	54	39	21	15	10	7.0	2	1.0	43	31	10	7.0	140	35
*Phonological condition*														
Valid	64	45.39	16	11.35	10	7.09	4	2.84	38	26.95	9	6.38	141	34
Control	75	47.77	14	8.92	17	10.83			44	28.03	7	4.46	157	18
*Semantic condition*														
Valid	65	43.62	17	11.41	16	10.74	1	0.67	45	30.20	5	3.36	149	26
Control	66	43.42	21	13.82	11	7.24			50	32.89	4	2.63	152	23

Note: % = proportion of errors.
